# Comparison of NLR and CRP in Identifying Organ Dysfunction-Based Severe Presentation of Acute Cholangitis in Patients with Choledocholithiasis: A Single-Center Retrospective Study

**DOI:** 10.3390/medicina62050815

**Published:** 2026-04-24

**Authors:** Cristina Angelescu, Matei-Alexandru Cozma, Radu Bogdan Mateescu, Ioana Anca Bădărău

**Affiliations:** 1 Faculty of Medicine, Carol Davila University of Medicine and Pharmacy, 050474 Bucharest, Romania; matei.cozma@gmail.com (M.-A.C.); bogmateescu@gmail.com (R.B.M.); anca.badarau@umfcd.ro (I.A.B.); 2 Department of Gastroenterology and Hepatology, Colentina Clinical Hospital, 020125 Bucharest, Romania

**Keywords:** acute cholangitis, choledocholithiasis, inflammation markers, neutrophil-to-lymphocyte ratio (NLR), C-reactive protein (CRP)

## Abstract

*Background and Objectives:* Early identification of patients at risk for severe forms of acute cholangitis (AC) is crucial for guiding prompt therapeutic interventions. In addition to traditional biomarkers such as C-reactive protein (CRP), newer indices like the neutrophil-to-lymphocyte ratio (NLR) are cost-effective and easy-to-use diagnostic tools. In the context of limited evidence from Eastern European populations, this study aimed to compare the performance of NLR and CRP in identifying organ dysfunction-based severe presentation of acute cholangitis according to a modified TG18-derived classification. *Materials and Methods:* We conducted a retrospective study including patients admitted to Colentina Clinical Hospital between January 2021 and September 2025. Demographic, clinical, and laboratory data were collected from medical records, with a focus on admission inflammatory biomarkers: NLR and CRP. Symptom onset-to-presentation time was not recorded. Patients were stratified by disease severity according to a modified organ dysfunction-based severity classification, and comparative statistical analysis, including Receiver Operating Characteristic (ROC) curve analysis, was performed to assess the diagnostic performance and optimal cut-off values of both biomarkers for severe clinical presentation. *Results:* A total of 364 patients were included, with a median age of 68 years (IQR: 60–78), and a slight female predominance (55.5%). Of these, 231 were classified as non-severe and 133 as severe. Both CRP and NLR were significantly higher in severe cases (*p* = 0.042 and *p* = 0.018, respectively. Despite statistical significance, CRP showed limited discriminatory ability, whereas NLR showed only a marginal numerical advantage. ROC analysis showed poor discriminatory ability for both CRP (AUC = 0.564, 95% CI 0.503–0.624, *p* = 0.042) and NLR (AUC = 0.574, 95% CI 0.513–0.635, *p* = 0.018) in predicting severe AC incidence. The difference between AUCs was minimal (ΔAUC = 0.010, 95% CI −0.062 to 0.082, *p* = 0.78, DeLong test). *Conclusions:* Both CRP and NLR showed limited discriminatory performance in identifying organ dysfunction-based severe presentation of acute cholangitis. The small, non-significant difference between the two markers does not support a meaningful comparative advantage, and neither biomarker appears suitable for reliable early risk stratification on its own.

## 1. Introduction

Acute cholangitis (AC) is a potentially life-threatening disorder characterized by bacterial infection of the biliary tree, typically ascending from the small intestine. The most frequent etiology is choledocholithiasis, when calculi within the common bile duct (CBD) precipitate partial or complete obstruction of the biliary system, thereby facilitating infection, and is responsible for almost 85% of cases of acute cholangitis [1,2]. AC is a relatively uncommon condition, with fewer than 200,000 cases being reported annually in the United States of America (USA). The mean age of affected individuals ranges from 50 to 60 years, with no significant difference in prevalence between males and females. Among hospitalized patients with gallstone disease in the USA, approximately 6% to 9% are diagnosed with AC [1].

Accurate diagnosis and severity grading remain essential, as mortality rates escalate dramatically from zero in mild cases to 20% in severe acute cholangitis, with early biliary drainage reducing 30-day mortality by over 50% in grade II patients [3]. Currently, the Tokyo Guidelines 2018 (TG18) represent the international standard for diagnosis and severity assessment. Overall, mortality rates remain at up to 15%, even with the availability of broad-spectrum antimicrobial therapy and advances in emergency endoscopic biliary tract decompression techniques [4].

Choledocholithiasis occurs in approximately 8–16% of patients with symptomatic gallstones and, with a growing incidence in recent years, it currently represents the second most common admission diagnosis across all gastrointestinal conditions in the USA [5]. Hospitalization rates for choledocholithiasis and AC rose notably in the USA between 2005 and 2014 [6]. This trend reflects the growing prevalence of gallstone disease in the USA, as well as a greater need for inpatient management of these conditions [6].

The neutrophil-to-lymphocyte ratio (NLR) represents the balance between the innate (neutrophils) and the adaptive immune response (lymphocytes), and it increases significantly in infections and sepsis, and sometimes precedes changes in other traditional markers [7]. NLR is widely recognized as a simple, inexpensive indicator of systemic inflammation in many acute abdominal disorders. Elevated NLR values have been linked to greater severity in acute cholecystitis, helping to identify patients who may need more aggressive treatment [8]. In acute appendicitis, higher NLR on admission correlates with complicated disease, perforation risk, and postoperative infectious complications, making it a useful adjunct when imaging is unavailable [9]. The ratio also predicts bowel ischemia in non-strangulated adhesive small-bowel obstruction, assisting early decision-making about surgical intervention [10]. Across these conditions, NLR provides clinicians with rapid prognostic insight without the need for costly tests.

In Romania, recent multicenter studies have highlighted the growing clinical significance of AC in tertiary referral centers, with the ARISE trial documenting 498 patients across three major endoscopy units and revealing concerning patterns of multidrug resistance and severity distribution [11]. Romanian healthcare data demonstrates distinct microbial profiles and antimicrobial resistance patterns, with 60.1% of biliary cultures being positive and 17.4% of isolates showing multidrug resistance, particularly in post-cholecystectomy patients. These regional epidemiological characteristics underscore the importance of developing locally validated predictive tools for severity assessment in Romanian tertiary care settings [12].

The rationale for this study arose from a series of published data indicating that NLR is a simple, reproducible, and cost-effective indicator of systemic inflammatory status and a stronger predictor of complication development than other inflammatory markers (CRP, Erythrocyte Sedimentation Rate—ESR). Recent clinical studies also demonstrated the superiority of NLR in finding severe forms, complications, and septic shock compared with CRP or total leukocyte count [7].

The importance of this study is further emphasized by the fact that, to our knowledge, very little data have been reported on cohorts from Romania or Eastern Europe regarding this topic. Given the known geographical variability in the epidemiology, clinical presentation, and outcomes of patients with AC, it is essential to investigate how the disease manifests and evolves within different healthcare settings and real-world medical practices. Generating local evidence not only contributes to a more accurate understanding of disease burden in our population but also provides valuable insights that may inform clinical decision-making and the adaptation of international guidelines to regional contexts.

Our aim is to compare the diagnostic performance of NLR versus C-reactive protein (CRP) to reflect the severity of acute cholangitis, based on a modified TG18-derived classification, and to explore their potential role as adjuncts to existing severity assessment strategies in patients with choledocholithiasis. In addition, we sought to determine optimal cutoff values for both biomarkers and explore their potential role in early risk stratification. By addressing these objectives, this research provides clinically relevant data from a Romanian tertiary care center and adds to the limited evidence available from this region.

## 2. Materials and Methods

### 2.1. Study Design

To test our hypothesis, we performed a retrospective observational cohort study using medical records of patients admitted to the Gastroenterology Department of Colentina Clinical Hospital, a tertiary referral center in Bucharest with a confirmed diagnosis of choledocholithiasis. The study included cases treated between January 2021 and September 2025; however, data extraction and analysis were initiated only after institutional ethical approval was granted. During this period, 631 patients were diagnosed with choledocholithiasis. Of these, 364 patients met the TG18 diagnostic criteria for AC at the time of admission.

### 2.2. Patient Selection

**Figure 1:**
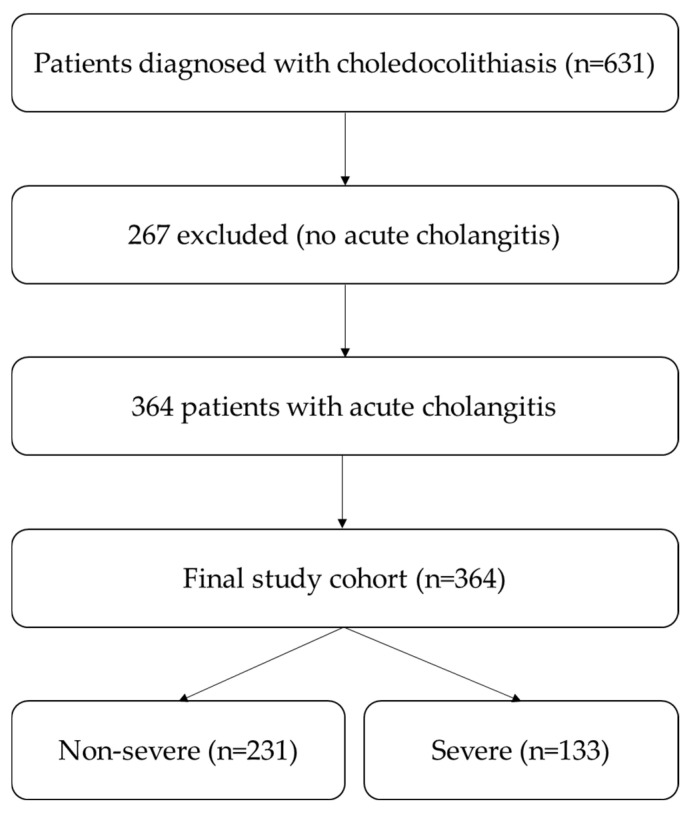
Flowchart of patient selection and study population.

All eligible patients meeting the inclusion criteria during the study period were consecutively included in the analysis. Patients were considered eligible if they were ≥18 years old, had a diagnosis of acute cholangitis, and had confirmed choledocholithiasis, established either before admission at referring centers or during hospitalization in our clinic, based on clinical symptoms, laboratory abnormalities (elevated liver enzymes and bilirubin), and imaging findings. Exclusion criteria included the presence of other biliary tract pathologies, such as malignancy or benign strictures of the CBD, which could potentially influence clinical management. Patients were also excluded if they had incomplete medical data, if they had a history of abdominal surgery that altered biliary anatomy, if they had received antibiotic therapy prior to admission to the ward, or if they had concurrent neoplasms or other hematologic disorders, as well as those receiving corticosteroid therapy. Another important aspect worth noting is that the selected patients did not present with any other infectious pathologies at the time of admission, which could otherwise have contributed to elevated inflammatory markers. This point is particularly relevant because the patient selection period also overlapped with the COVID-19 pandemic. In addition, all patients underwent pre-admission screening, including testing for COVID-19, prior to hospitalization. The inclusion and exclusion criteria are summarized in Table 1, and the patient selection process is illustrated in Figure 1.

### 2.3. Diagnostic Protocol

AC was diagnosed according to the TG18 algorithm [13]. Patients were classified as having suspected cholangitis if they presented with systemic inflammation (fever or chills and/or abnormal WBC count or elevated CRP) in association with either cholestasis (defined as jaundice or abnormal liver function tests, with thresholds of total bilirubin (TB) ≥ 2 mg/dL, alkaline phosphatase (ALP) > 150 U/L, gamma glutamyl transferase (GGT) > 75 U/L, or aspartate aminotransferase (AST)/ alanine aminotransferase (ALT) > 60 U/L) and imaging evidence of biliary obstruction. A definite diagnosis required the simultaneous presence of all three diagnostic domains: systemic inflammation, cholestasis, and imaging findings.

Severity of AC was assessed according to available TG18-derived organ dysfunction parameters, rather than full TG18 grading. The decision to adapt the original criteria was determined by the retrospective nature of our database, in which complete clinical information required for the standard TG18 severity grading was not available. Specifically, for Grade III (severe cholangitis), data on cardiologic, neurologic, and pulmonary dysfunction were missing. Consequently, organ failure was defined based on the parameters that were consistently available: serum creatinine, platelet count, and INR. This modified grading system was developed to allow a uniform and objective assessment of severity within the constraints of the available dataset, while maintaining the core principle of identifying patients with organ dysfunction. Therefore, severe disease was defined based on consistently available markers, including renal impairment (creatinine > 2.0 mg/dL), coagulopathy (INR > 1.5), or thrombocytopenia (<100,000/µL). Moderate disease was defined by the presence of at least two of the following criteria: abnormal white blood cell count, high-grade fever, age ≥ 75 years, total bilirubin ≥ 5 mg/dL, or hypoalbuminemia. Patients who did not fulfill the criteria were classified as mild. For the purposes of this analysis, patients were divided into severe (presence of organ dysfunction) and non-severe (mild to moderate disease without organ dysfunction) categories.

Initial blood samples were systematically drawn within a maximum time frame of two hours following patient admission. However, the time from symptom onset to hospital presentation was not systematically recorded and, therefore, could not be accounted for in the analysis. Consistent with the institutional management protocol, the initiation of empirical broad-spectrum antibiotic therapy was subsequently performed only after the results of these initial laboratory analyses were made available.

### 2.4. Data Acquisition and Study Variables

All variables were extracted from the patients’ medical records, including admission charts and hospital discharge summaries. To ensure confidentiality, all data were fully anonymized prior to analysis, and no personally identifiable information was retained. The dataset included a broad range of demographic, clinical, biological, and imaging variables. Demographic data comprised age, sex, and lifestyle-related factors, including alcohol consumption and tobacco use. Clinical presentation at admission was documented in terms of abdominal pain, jaundice, nausea or vomiting, fever, and chills. Biological parameters included liver function enzymes (AST, ALT, ALP, GGT, and TB), renal function tests (creatinine), hematological indices (WBC count, platelet count, neutrophil and lymphocyte counts), coagulation profile (International Normalized Ratio—INR), and CRP. Imaging findings obtained by ultrasonography, Computer Tomography (CT), or Magnetic Resonance Cholangiopancreatography (MRCP) were reviewed and included details on CBD dilatation and stone size.

### 2.5. Statistical Analysis

Statistical analyses were performed using SPSS version 26.0 (IBM Corp., Armonk, NY, USA). Categorical variables, such as sex, smoking, alcohol consumption, fever or chills, abdominal pain, and comorbidities, were expressed as frequencies and percentages and compared between groups using Pearson’s χ^2^ test. The normality of continuous variables, including age, CRP, NLR, WBC, TB, INR, creatinine, platelet count, and hepatic enzymes, was assessed using the Shapiro–Wilk test. Normally distributed data were presented as mean ± standard deviation (SD) and compared between groups with the independent-samples *t*-test, whereas non-normally distributed variables were reported as median and interquartile range (IQR) and compared using the Mann–Whitney U test. To evaluate the performance of inflammatory markers, receiver operating characteristic (ROC) curves were constructed for CRP and NLR, and the area under the curve (AUC) was calculated with corresponding 95% confidence intervals. Optimal cut-off values for discrimination were determined using Youden’s index, with reporting of the associated sensitivity and specificity. All statistical tests were two-sided, and a *p*-value of <0.05 was considered statistically significant.

## 3. Results

Out of 631 patients who were diagnosed with choledocholithiasis, 267 patients had no evidence of cholangitis at the time of admission, whereas 364 patients met diagnostic criteria for acute cholangitis according to the TG18. Our study cohort comprised patients who were diagnosed with acute cholangitis at the moment of admission.

In our group, 162 patients (44.5%) were male and 202 (55.5%) were female. The sex distribution was similar between patients with non-severe and severe presentation of AC (44.6% vs. 44.4% male, *p* = 0.97). The median age of patients included in the study was 68 years [IQR: 60–78]. No significant differences were observed between patients with non-severe and severe clinical presentation of AC (68 years [IQR: 58–79] vs. 70 years [IQR: 63–78], *p* = 0.20). The main differences between the two groups in terms of main demographic characteristics, lifestyle factors, and laboratory findings are detailed in Table 2, below.

TB values were significantly higher in patients with severe clinical presentation compared to those with non-severe forms (1.5 mg/dL vs. 1.9 mg/dL, *p* = 0.042). Serum AST and GGT values were significantly higher in the severe group compared to the non-severe group (*p* = 0.028 and *p* = 0.003, respectively). ALP also showed a highly significant increase in the severe group (*p* < 0.001). In contrast, ALT did not differ significantly between groups (*p* = 0.769).

As expected, based on the definition of the modified organ dysfunction-based severity classification, platelet count differed significantly (*p* < 0.001) between patients with and without severe presentation of acute cholangitis, with median values of 138 [63.5–240] and 272 [196–357], respectively. Similarly, INR values were significantly higher in patients with severe presentation compared to those without (1.52 vs. 1.24, *p* < 0.001). The Shapiro–Wilk test indicated that creatinine values were normally distributed in patients without severe presentation (*p* = 0.117), but not in those with severe presentation (*p* < 0.001). Therefore, the Mann–Whitney U test showed significantly higher creatinine levels in patients with severe presentation (1.13 mg/dL) compared to those without (1.03 mg/dL, *p* = 0.009).

**Figure 2:**
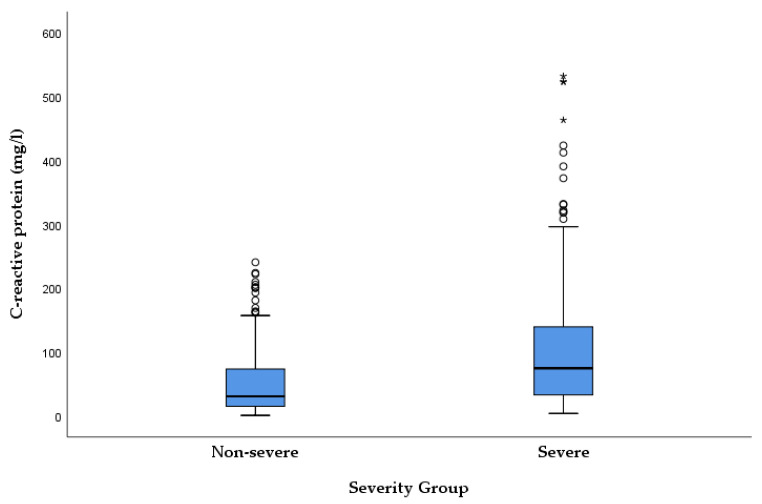
Distribution of CRP values in patients with AC, stratified according to the modified organ dysfunction-based severity classification (Mann–Whitney U test, *p* = 0.042). The central line represents the median, the box indicates the interquartile range (IQR), and whiskers extend to 1.5 × IQR. Circles denote outliers, while asterisks (*) indicate extreme values (>3 × IQR).

**Figure 3:**
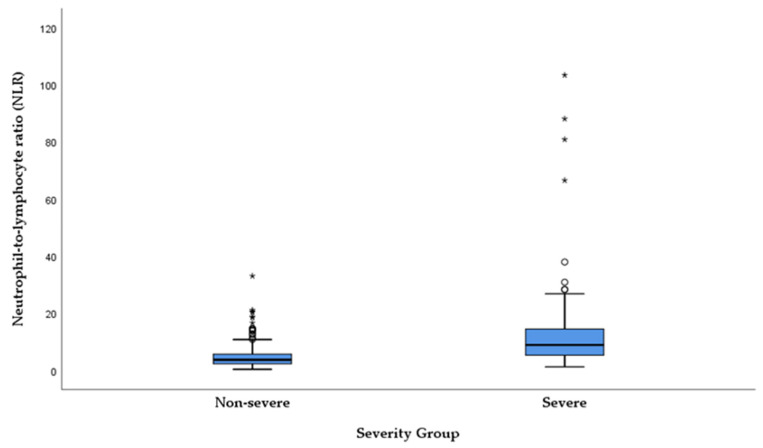
Distribution of NLR values in patients with AC, stratified according to the modified organ dysfunction-based severity classification (Mann–Whitney U test, *p* = 0.018). The central line represents the median, the box indicates the interquartile range (IQR), and whiskers extend to 1.5 × IQR. Circles denote outliers, while asterisks (*) indicate extreme values (>3 × IQR).

In the studied cohort, CRP values were significantly higher in patients with severe presentation compared to those with non-severe clinical manifestation (median = 42, IQR = [18–83] vs. 30 [14–74], *p* = 0.042). Similarly, NLR values were elevated in the severe group, with the difference being highly statistically significant (median = 4.5 [2.7–7.4] vs. 3.7 [2.3–5.7], *p* = 0.018). When compared directly, the discriminatory ability in distinguishing patients with severe presentation of acute cholangitis from those without, based on the modified classification, was similar between the two markers, with NLR showing only a slight numerical advantage. Figure 2 and Figure 3 provide a visual representation of CRP and NLR values, stratified by severity (severe vs. non-severe clinical presentation based on the modified classification).

**Figure 4:**
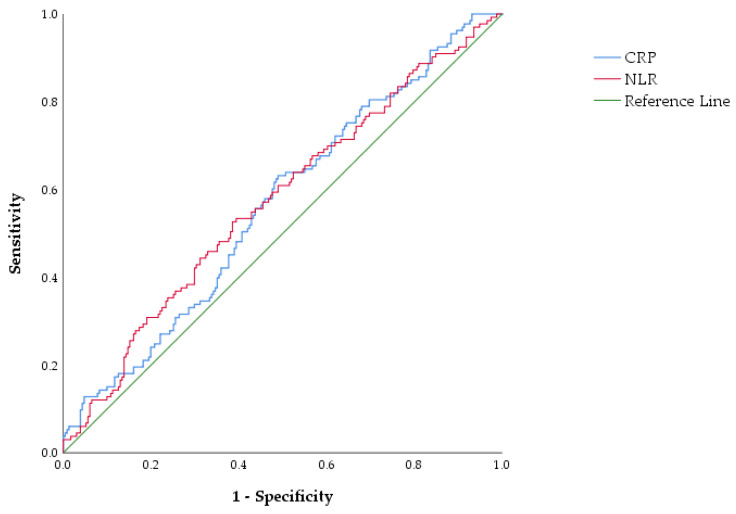
ROC curves for CRP and NLR in differentiating severe from non-severe clinical presentation of AC. CRP: AUC = 0.564 (95% CI 0.503–0.624, *p* = 0.042); NLR: AUC = 0.574 (95% CI 0.513–0.635, *p* = 0.018); ΔAUC = 0.010 (95% CI −0.062–0.082, *p* = 0.78, DeLong test).

ROC curve analysis was performed to assess the ability of CRP to discriminate between patients with and without severe presentation of acute cholangitis, as defined by the modified classification. The area under the curve (AUC) for CRP was 0.564 (95% CI: 0.503–0.624, *p* = 0.042). The ROC analysis for NLR yielded an AUC of 0.574 (95% CI 0.513–0.635, *p* = 0.018), indicating very poor discriminatory ability, with performance close to chance level despite statistical significance. As shown in Figure 4, both biomarkers demonstrated similarly limited performance in identifying severe presentation of acute cholangitis (modified classification).

ROC analysis identified an optimal cutoff for CRP at 30.9 mg/L, yielding a sensitivity of 63.2% and a specificity of 51.1% (Youden index = 0.143). This means that CRP correctly identified approximately two-thirds of severe cases, but also misclassified nearly half of the non-severe patients as positive. The positive predictive value was 42.3%, indicating that fewer than half of the patients with elevated CRP above this threshold were actually severe. Conversely, the negative predictive value was 70.7%, showing that a normal CRP below the threshold could exclude severity in about seven out of ten patients. The likelihood ratios (LR+ = 1.29; LR− = 0.73) were close to 1, confirming that CRP provided only a minimal change in the probability of severe disease. At a high-sensitivity threshold (≥4.13 mg/L), sensitivity reached 97.7% with poor specificity (8.7%). Conversely, at a highly specific threshold (≥133 mg/L), specificity was 90.0%, but sensitivity dropped to 15.0%.

For NLR, the optimal cutoff identified by Youden’s index was 4.51, yielding a sensitivity of 52.6% and a specificity of 61.5% (Youden index = 0.142). This indicates that NLR correctly classified just over half of the severe cases, while misclassifying nearly 40% of the non-severe patients as positive. The positive predictive value was 44.0%, showing that fewer than half of the patients with an elevated NLR above the threshold were truly severe. The negative predictive value was 69.3%, meaning that approximately seven out of ten patients with a low NLR were indeed non-severe. The likelihood ratios (LR+ = 1.37, LR− = 0.77) were close to 1, suggesting that NLR provides only a minimal shift in the post-test probability of severe disease. At a sensitivity-oriented threshold (≥1.32), sensitivity was 97.7%, but specificity dropped to 5.2%. At a specificity-oriented threshold (≥9.85), specificity was 90.0% while sensitivity decreased to 12.8%. The diagnostic performance and the threshold values for the two markers are summarized in Table 3 and Table 4.

ROC analysis showed poor discriminatory ability for both CRP (AUC = 0.564, 95% CI 0.503–0.624, *p* = 0.042) and NLR (AUC = 0.574, 95% CI 0.513–0.635, *p* = 0.018) in identifying severe presentation of acute cholangitis, as defined by the modified classification. The difference between AUCs was small (ΔAUC = 0.010, 95% CI −0.062–0.082, *p* = 0.78, DeLong test). Figure 4 provides a visual representation of these findings.

## 4. Discussion

In recent years, additional inflammatory biomarkers have been explored for their potential role in assessing disease severity in acute biliary infections. Procalcitonin has shown promising results in predicting severe infection and septic complications [14], while interleukin-6 (IL-6), an early pro-inflammatory cytokine, has been associated with the intensity of systemic inflammatory response [15]. Furthermore, butyrylcholinesterase has been proposed as a negative acute-phase reactant, with decreased levels reflecting systemic inflammation and poorer clinical outcomes [16]. However, the role of these biomarkers in acute cholangitis remains incompletely defined, and they are not currently incorporated into standard diagnostic or severity assessment frameworks. Despite these developments, their routine clinical use is limited by availability, cost, and lack of standardized thresholds. In contrast, NLR and CRP remain widely accessible and inexpensive, supporting their continued evaluation in clinical practice.

While previous research has acknowledged the role of both NLR and CRP as indicators of systemic inflammation, conflicting evidence has persisted regarding which marker offers superior predictive performance specifically for severe outcomes in AC. Our findings suggest that these two readily accessible markers have similarly limited ability to differentiate between patients with and without modified organ dysfunction-based severe presentation. Although NLR showed a numerically higher AUC compared to CRP, this difference was not statistically significant and did not translate into meaningful clinical superiority, given the overall poor discriminatory performance of both markers.

Limitations exist in NLR interpretation. Factors such as corticosteroid use, chemotherapy, and underlying hematologic disorders can affect neutrophil and lymphocyte counts independently of infection [17], and that is why patients with a history of pre-admission pharmacological interventions, such as antibiotic therapy or corticosteroid administration, were excluded from the study cohort. Antibiotic therapy influences NLR by controlling the underlying infection, which subsequently reduces the inflammatory response. As effective antibiotic treatment suppresses bacterial load, neutrophil count decreases, and lymphocyte count recovers, leading to a decline in NLR values and potentially masking the true severity of the acute inflammatory process [18].

We need to address the fact that even when blood sampling is standardized within the first two hours of hospital admission, as per the protocol in our department, significant variability in the time elapsed from symptom onset to presentation represents a critical confounding factor in retrospective biomarker studies of acute cholangitis severity. This pre-hospital delay introduces substantial heterogeneity, as biomarker levels, particularly CRP and NLR, follow dynamic trajectories that evolve with disease progression and may peak at different intervals following the initial inflammatory insult. Recent research underscores that time factors, including delays in presentation and admission, directly influence patient outcomes and biomarker profiles in acute cholangitis, thereby complicating the interpretation of admission values as true baseline severity indicators [7,19,20]. This limitation is particularly relevant in retrospective analyses where the exact symptom-to-admission interval is often imprecisely documented, potentially leading to misclassification of disease severity and overestimation of biomarker predictive accuracy [7,21].

Literature cohorts show consistent patterns in AC severity distribution, with severe cases representing a significant minority while mild to moderate cases constitute the majority. A large national validation study of the Tokyo Guidelines 2018 involving 137,100 patients found that 68.09% had non-severe (mild/moderate) cholangitis while 31.91% had severe cholangitis [22]. A more recent 2024 study corroborated this distribution, reporting that 62.5% of cases were mild/moderate compared to 37.5% severe [23]. Direct comparison with previously published studies is limited, as full TG18 severity grading was not applied; nevertheless, the relatively high proportion of cases with modified organ dysfunction-based severe presentation (36.5% in our cohort) may reflect the specific context of a tertiary referral center, where elderly patients with multiple comorbidities and more severe forms are overrepresented, and where many cases arrive as transfers already selected for greater complexity. In addition, patients with chronic liver disease or chronic kidney disease may present baseline INR values > 1.5 or elevated creatinine levels, which may have led to an overestimation of severe cases. Although this represents a limitation, TG18 criteria impose strict adherence to these thresholds, and most international validation studies do not adjust for chronic disease. Several potential biases must also be acknowledged: severity bias due to the high mean age (66.5 years, with 30.6% ≥75 years) and hospitalization of predominantly moderate-to-severe cases, referral bias given the inclusion of complicated referrals from other hospitals, retrospective selection and survival biases, since mild cases, early discharges, or rapidly fatal cases may be underrepresented. Taken together, these factors suggest that the proportion of severe cases in our dataset likely reflects case mix and retrospective limitations rather than a true epidemiological shift.

Several studies have suggested that NLR may outperform CRP in recognizing disease severity, although results have been inconsistent across different cohorts. However, our findings did not confirm this observation. NLR consistently outperformed CRP across multiple studies, with a 2023 analysis showing that NLR increases significantly with cholangitis severity while maintaining better discrimination power than traditional inflammatory markers [24]. In a landmark study of 206 patients, NLR showed superior association accuracy compared to CRP, achieving an AUC of 0.87 for severe acute cholangitis compared to CRP’s AUC of 0.74. The optimal NLR cutoff of 15.24 provided 85% sensitivity and 79% specificity for identifying severe cholangitis [7]. A 2024 study confirmed NLR’s superior performance with an AUC of 0.759 and optimal cutoff of 5.595, establishing it as the best inflammatory marker for severity recognition [23]. Combined biomarker approach using both CRP ≥ 10.3 mg/dL and NLR ≥ 6.7 achieved the highest positive predictive value of 90% for acute suppurative cholangitis [2].

Although several studies have suggested a potential advantage of NLR over CRP in predicting disease severity, our findings did not support this observation. In our cohort, both NLR and CRP demonstrated poor discriminatory performance identifying patients with modified organ dysfunction-based severe presentation of acute cholangitis, with AUC values close to 0.5, indicating performance near chance level. While statistically significant, these results have limited clinical relevance and do not support the use of either biomarker as a reliable standalone tool for severity assessment. In addition, likelihood ratios for both markers were close to 1, suggesting minimal impact on post-test probability and further underscoring their limited clinical utility. These discrepancies compared with previous reports may reflect differences in patient populations, disease severity definitions, or study design. Overall, our findings suggest that the prognostic value of NLR and CRP is limited and this variability highlights the need for cautious interpretation and reinforces the importance of external validation across different clinical settings. Our optimal cutoff value for NLR (4.51; Se 52.6%, Sp 61.5%) was similar to those reported in diagnostic-oriented studies (around 5.3) but considerably lower than the thresholds proposed for severity prediction in other cohorts. For instance, Lee et al. (2022, Korea, *n* = 206) found that NLR increased with disease severity, reporting an AUC of 0.87 for severe AC—significantly outperforming CRP (AUC = 0.74), and proposed an optimal cutoff of 15.24 (Se 85%, Sp 79%) [7]. They also highlighted the potential utility of serial NLR monitoring (admission vs. day 2) to track progression [7]. Likewise, Li et al. (2024, *n* = 139) reported an AUC of 0.759 for NLR alone with a cutoff around 5.6, while their combined index (PNS obtained from serum albumin level and total lymphocyte count that captures a patient’s nutritional and immune status) reached an AUC of 0.853, outperforming single markers [23]. This suggests that considering both inflammation and nutritional status together yields a better risk stratification tool for patients with severe AC.

For CRP, our optimal cutoff of 31 mg/L (Se 63.2%, Sp 51.1%) demonstrated only modest ability to discriminate between patients with and without severe presentation according to the modified classification. This aligns with the findings in the literature, of Ye et al. (2023, n = 293), who reported that CRP differentiated mild from moderate acute cholangitis with an AUC of 0.66, whereas procalcitonin performed better in distinguishing mild from severe cases (AUC = 0.80) [25]. Similarly, Beliaev et al. (2018) found that CRP ≥ 23.5 mg/L had good diagnostic but limited prognostic value in acute cholangitis, supporting our observation that CRP, although elevated in severe disease, lacks sufficient discriminative power for accurate severity stratification [26]. The modest balance between specificity and sensitivity observed in our study suggests that these markers should be integrated into composite scores or combined with clinical parameters rather than used in isolation. Although our findings are in line with previous reports, comparisons should be made with caution due to differences in severity classification across studies. Moreover, thresholds achieving very high sensitivity or specificity represent clinically unbalanced scenarios and should not be interpreted as supporting rule-out or rule-in strategies, particularly in the context of overall poor discrimination. Finally, the proposed cutoff values for both CRP and NLR should be interpreted with caution, given the absence of external validation and the potential for threshold overfitting.

### Study Limitations

This study has several limitations that should be considered when interpreting the findings. First, its retrospective single-center design may limit the applicability of the results and introduces the possibility of selection bias, although consecutive patient inclusion was ensured.

Second, the severity classification was based on a modified set of TG18-derived parameters, due to incomplete availability of all criteria required for standard grading. As a result, the definition of severe disease does not fully correspond to the conventional TG18 framework, and some degree of misclassification cannot be excluded. In addition, certain laboratory parameters, such as creatinine, INR, and platelet count, were incorporated into the definition of severity, which may have influenced comparisons between groups.

Another important limitation relates to the derivation of optimal cutoff values using Youden’s index without external validation. This data-driven approach carries a risk of threshold overfitting and optimistic bias, and therefore the proposed cutoffs should be considered exploratory and interpreted with caution until validated in independent cohorts [27].

Furthermore, although blood sampling was standardized at admission, the time from symptom onset to hospital presentation was not precisely documented. Given the dynamic nature of inflammatory biomarkers, this variability may have influenced their measured values and contributed to the limited discriminatory performance observed. This represents a key limitation, as it may have substantially affected the diagnostic accuracy of the evaluated biomarkers.

Finally, the relatively low AUC values and likelihood ratios close to 1 highlight the limited ability of these biomarkers to reliably discriminate between severe and non-severe cases when used in isolation, supporting the need for integrated clinical assessment and more robust predictive models.

## 5. Conclusions

This study provides a comparative evaluation of NLR and CRP in identifying organ dysfunction-based severe presentations of acute cholangitis, according to a modified TG18-derived classification. Although both markers were significantly elevated in severe cases, their discriminatory performance was poor, with limited clinical utility for risk stratification when used as standalone markers. No significant difference was observed between NLR and CRP, with NLR showing only a numerically higher performance. The proposed cutoff values should be considered exploratory, as they were derived from a single-center retrospective cohort and were not externally validated. These findings are consistent with current guidelines, supporting the use of inflammatory markers as adjuncts rather than replacements for clinical and biochemical assessment of organ dysfunction. Importantly, these results offer a pragmatic perspective on the real-world performance of commonly used inflammatory biomarkers, helping to refine expectations regarding their role in clinical practice. Beyond the direct comparison between NLR and CRP, the present study provides additional value by offering real-world data from a Romanian tertiary care center, a setting that remains underrepresented in the current literature. Future multicenter and prospective studies are required to validate these findings and to explore more robust predictive models, including combined biomarker approaches.

## Figures and Tables

**Table 1:** Inclusion and exclusion criteria.

Inclusion Criteria	Exclusion Criteria
Age ≥ 18 years	Age ≤ 18 years
Confirmed choledocholithiasis	Incomplete medical records
Diagnosis of acute cholangitis	Presence of other biliary tract pathologies (benign strictures, malignancy, etc.)
	Prior surgery altering biliary anatomy
	Antibiotic therapy prior to admission
	Hematologic disorders
	Corticosteroid therapy
	Other concurrent infections

**Table 2:** Baseline demographic, clinical, and laboratory characteristics of patients with severe vs. non-severe clinical presentation of AC.

Characteristic	Total	Non-Severe Group	Severe Group	*p*-Value
Total, n (%)	364 (100%)	231 (63.5%)	133 (36.5%)	
**Demographic data**				
Sex				0.97
Male	162 (44.5%)	103 (44.6%)	59 (44.4%)	
Female	202 (55.5%)	128 (55.4%)	74 (55.6%)	
Age, median [IQR]	68 [60–78]	68 [58–79]	70 [63–78]	0.20
**Lifestyle**				
Smokers	105 (28.8%)	72 (31.2%)	33 (24.8%)	0.20
Drinkers	284 (78%)	179 (77.5%)	105 (78.9%)	0.75
**Symptoms**				
Fever	103 (28.3%)	58 (25.1%)	45 (33.8%)	0.08
Abdominal pain	240 (65.9%)	152 (65.8%)	88 (66.2%)	0.94
Nausea/vomiting	117 (32.1%)	77 (33.3%)	40 (30.1%)	0.52
**Comorbidities**				
Diabetes mellitus	64 (17.6%)	42 (18.2%)	22 (16.5%)	0.69
Obesity	75 (20.6%)	42 (18.2%)	33 (24.8%)	0.13
Cardiovascular comorbidities	268 (73.6%)	171 (74%)	97 (72.9%)	0.82
**Biological**				
CRP (mg/L)	36 [15–75]	30 [14–74]	42 [18–83]	0.042
NLR	3.9 [2.4–6.2]	3.7 [2.3–5.7]	4.5 [2.7–7.4]	0.018
WBC (×1000/uL)	9.2 [7.1–11.5]	9.2 [7–11]	9.2 [7.3–12]	0.20
PLT (×1000/uL)	229 [150–325]	272 [196–357]	138 [63.5–240]	<0.001
TB (mg/dL)	1.6 [0.7–4]	1.5 [0.65–3.5]	1.9 [0.83–5]	0.042
INR	1.28 [1.1–1.44]	1.24 [1.1–1.35]	1.52 [1.2–1.6]	<0.001
Creatinine (mg/dL)	1.06 [0.6–1.3]	1.03 [0.6–1.26]	1.13 [0.62–1.45]	0.009
TGO (U/L)	65 [38–121]	60 [36–109]	75 [42–140]	0.028
TGP (U/L)	103 [52–173]	104 [50–187]	102 [53–166]	0.77
ALP (U/L)	266 [173–447]	239 [158–344]	369 [201–603]	<0.001
GGT (U/L)	467 [235–828]	437 [228–746]	614 [285–1023]	0.003

**Table 3:** Diagnostic performance of CRP and NLR in assessing severe clinical presentation of AC.

Marker	AUC (95% CI)	*p*-Value	Optimal Cutoff	Sensitivity %	Specificity %	PPV %	NPV %	LR+	LR−
CRP	0.564 (0.503–0.624)	0.042	31	63.2	51.1	42.3	70.7	1.29	0.73
NLR	0.574 (0.513–0.635)	0.018	4.51	52.6	61.5	44	69.3	1.37	0.77

**Table 4:** Cutoff thresholds for CRP and NLR.

Marker	Cutoff	Sensitivity %	Specificity %	Note
CRP	31 mg/L	63.2	51.1	Optimal (Youden)
	4.13 mg/L	97.7%	8.7%	Triage (Sens ≥ 90%)
	133 mg/L	15.0	90.0	Rule-in (Spec ≥ 90%)
NLR	4.51	52.6	61.5	Optimal (Youden)
	1.32	97.7	5.2	Triage (Sens ≥ 90%)
	9.85	12.8	90	Rule-in (Spec ≥ 90%)

## Data Availability

The raw data supporting the conclusions of this article will be made available by the authors on request.

## Abbreviations

The following abbreviations are used in this manuscript:

AC	Acute cholangitis
ALP	Alkaline phosphatase
ALT	Alanine aminotransferase
AST	Aspartate aminotransferase
AUC	Area under the curve
CBD	Common bile duct
CRP	C-reactive protein
CT	Computed tomography
ERCP	Endoscopic retrograde cholangiopancreatography
ESR	Erythrocyte sedimentation rate
GGT	Gamma-glutamyl transferase
INR	International normalized ratio
IQR	Interquartile range
MRCP	Magnetic resonance cholangiopancreatography
NLR	Neutrophil-to-lymphocyte ratio
OR	Odds ratio
ROC	Receiver operating characteristic
SD	Standard deviation
TB	Total bilirubin
TG18	Tokyo Guidelines 2018
WBC	White blood cell
